# The prevalence and clinical context of antimicrobial resistance amongst medical inpatients at a referral hospital in Rwanda: a cohort study

**DOI:** 10.1186/s13756-024-01384-7

**Published:** 2024-02-22

**Authors:** Olivier Bizimungu, Peter Crook, Jean Félix Babane, Léopold Bitunguhari

**Affiliations:** 1 School of Medicine and Pharmacy, College of Medicine and Health Sciences, University of Rwanda, Kigali, Rwanda; 2 Department of Internal Medicine, Rwanda Military Hospital, Kanombe, KK 567 St, Kigali, Rwanda; 3 Institute for Infection and Immunity, St George’s, University of London, London, UK; 4 Department of Internal Medicine, University Teaching Hospital of Kigali, Kigali, Rwanda

**Keywords:** Antimicrobial resistance, Surveillance, Microbiology, Low- and middle-income countries (LMIC)

## Abstract

**Background:**

Antimicrobial resistance (AMR) is a growing global concern. AMR surveillance is a crucial component of the international response; however, passive surveillance of laboratory data is limited without corresponding patient-level clinical data. This study sought to examine the burden of AMR amongst medical inpatients in Rwanda, in the context of their clinical presentations and prior antibiotic exposures.

**Methods:**

This cohort study was conducted over a 9-month period at a tertiary referral hospital in Kigali, Rwanda. We enrolled 122 adult medical inpatients with a history of fever and a positive microbiological culture result. Data were collected regarding the clinical and microbiological aspects of their admission.

**Results:**

The most common diagnoses were urinary tract infection (n = 36, 30%), followed by pneumonia (n = 30, 25%) and bacteraemia (11 primary [9%] and 10 catheter-related [8%]). The most common pathogens were *E. coli* (n = 40, 33%) and *Klebsiella pneumoniae* (n = 36, 30%). The cohort were heavily antibiotic-exposed at the time of culture with 98% of patients (n = 119) having received an antibiotic prior to culture, with a median exposure of 3 days (IQR 2–4 days). Eighty patients (66%) were specifically prescribed ceftriaxone at the time of culture. Gram-negative organisms predominated (82% [100/122]) and exhibited high rates of resistance, with only 27% (21/77) being susceptible to ceftriaxone, 2.4% (2/82) susceptible to co-amoxiclav and 44% (8/18) susceptible to ciprofloxacin. Susceptibility amongst Gram-negatives was relatively preserved to amikacin (91%, 79/87) and imipenem (85%, 70/82). There were no cases of methicillin-resistant *Staphylococcus aureus* (0/12) or vancomycin-resistant enterococci (0/2). Discordant antibiotic therapy was significantly associated with in-hospital mortality (OR 6.87, 95%CI 1.80–45.1, *p* = 0.014).

**Conclusions:**

This cohort highlights high rates of resistance amongst Gram-negative organisms in Rwanda, including the presence of carbapenem resistance. Nonetheless, the detailed prescribing data also highlight the challenges of using routine laboratory data to infer broader AMR prevalence. The significant exposure to empiric broad-spectrum antibiotic therapy prior to culturing introduces a selection bias and risks over-estimating the burden of resistant organisms. Broadening access to microbiological services and active surveillance outside of teaching hospitals are essential to support national and international efforts to curb the growth of AMR in low-resource settings.

**Supplementary Information:**

The online version contains supplementary material available at 10.1186/s13756-024-01384-7.

## Introduction

Antimicrobial resistance (AMR) is now well recognised as one of the leading global health emergencies, with an estimated 1.27 million deaths attributed to bacterial AMR in 2019 [1, 2]. That figure is forecast to reach 10 million annual deaths by 2050 [3]. The burden of this mortality falls disproportionately on low-income countries, which also suffer from limited therapeutic options for resistant organisms, limited laboratory capacity and incomplete data [2].

A global, concerted effort to tackle AMR is underway, with a multidisciplinary One Health approach [4]. In Rwanda, a national action plan on AMR was published in 2021, outlining five strategic objectives to prevent and control the spread of AMR, of which the need for improved AMR surveillance was one [5].

Previous studies from teaching hospitals in Rwanda have reported high rates of AMR, particularly amongst Gram-negative infections where resistance to 3rd-generation cephalosporins has been consistently greater than 70% in the most recent studies [6, 7]. High rates of carriage of extended-spectrum beta-lactamase (ESBL) producing organisms have also been reported amongst patients and caregivers [8]. Meanwhile the rates of methicillin-resistant *Staphylococcus aureus* (MRSA) vary greatly between studies with a reported prevalence ranging from 2 to 82% [9, 10].

A significant challenge to understanding AMR in low-resource settings is the limited capacity for microbiological testing, particularly outside of teaching hospitals in capital cities. As such, the patients for whom AMR data are available may not be representative of the broader population. This is particularly true in Rwanda where patients typically follow a well-defined referral pathway, passing via health centres and district hospitals first before being referred on to teaching hospitals if they do not improve with first-line therapies. As such, cultures received in the laboratory are typically from patients who are already heavily antibiotic-exposed at the time of sampling, introducing a selection bias towards isolation of resistant organisms. This selection bias is increasingly recognised as a limitation of passive AMR surveillance, particularly where laboratory data are not linked to patient data [2]. In this study, we sought to define the prevalence of AMR in medical patients at a referral hospital in Kigali, Rwanda, and, crucially, to correlate this with patient-level data regarding clinical presentations, prior empirical antibiotic exposure and patient outcome.

## Materials and methods

### Study design, duration, setting and population

This was a prospective observational cohort study conducted at the University Teaching Hospital of Kigali (CHUK), a large tertiary referral hospital in Kigali, Rwanda, with 519 beds (of which 80 are medical and 22 are intensive care unit [ICU] beds). The study was conducted over a 9-month period from August 2021 to April 2022. Eligible patients included all adults (≥ 15 years) admitted to a medical ward or to the ICU under the medical team with: (i) a history of fever prior to or during admission; and (ii) a positive microbiological culture. Those with culture results that were deemed non-significant were excluded (see below). Eligible patients were identified daily by chart review by one of the study team (OB), who was working as an internist in the hospital.

### Patient consent

All participants gave informed, written consent to one of the study team (OB). Consent was obtained according to patient preference in Kinyarwanda, English or French. Participants were informed that taking part was voluntary and would not impact their clinical care.

### Data collection

Consenting participants were followed up prospectively until discharge, with data collected from their clinical notes, including: demographics, comorbidities, route of referral, antibiotic exposure both prior to and during admission, culture results (including identification of organism and susceptibility pattern), final diagnosis and outcome. Data were collected manually onto hardcopy case reference forms and later uploaded in anonymised format onto an online database (Epidata Software version 3.1, Odense, Denmark) [11].

### Microbiology

All specimens were processed routinely in the hospital’s laboratory according to its standard operating procedure. Details of this are as described by Munyemana et al*.,* 2022 [12]. In brief, Gram-negative organisms were identified using a combination of biochemical and other phenotypic tests (including Kligler’s Iron Agar (KIA), motility, indole, urea and citrate tests). API 20E was also used where necessary. Gram-positives were identified based on a combination of growth and appearance on blood and mannitol salt agar, response to catalase, coagulase, CAMP and/or bile eschulin tests and susceptibility to novobiocin, bacitracin or optochin. Susceptibility testing was performed by disc diffusion method (disc concentrations as described elsewhere) [12], with interpretation according to the 2021 Clinical and Laboratory Standards Institute (CLSI) guidelines (31st edition) [13]. Testing for resistance mechanisms (for example the production of extended-spectrum beta-lactamases) was not routinely performed and so data for these were not available.

Coagulase-negative staphylococci were not routinely speciated in the hospital laboratory during the study period and were therefore excluded as possible contaminants. Cultures with mixed growth from non-sterile sites were also excluded to reduce the risk of misidentifying commensal organisms as pathogens*.* Where an enrolled patient had more than one positive culture during admission, only their first episode of infection was included in the analysis.

When assigning diagnoses, bacteraemias were deemed to be catheter-related bloodstream infections where the patient had a central venous catheter in situ and no other source of the bacteraemia was identified; bacteraemia with Salmonella typhi was labelled as typhoid fever; all other bacteraemias were labelled as primary bacteraemias as there were no cases of bacteraemia with a positive culture from another source.

### Statistical analysis

Categorical data are presented as number (percentage) and continuous data as median (interquartile range [IQR]). Antimicrobial susceptibilities are presented as number of susceptible isolates (percentage of all isolates tested).

Binary logistic regression was used to assess the relationship between in-hospital mortality (outcome) and discordant antibiotic therapy (exposure), as well as the relationship between resistance (outcome; defined as resistance to any 3rd-generation cephalosporin) and site of infection acquisition (exposure; defined as fever onset ≥ 72 h after hospitalisation [hospital-acquired] or < 72 h from hospitalisation [community-acquired]). 95% confidence intervals were calculated using Wald's method of normal approximation.

All statistical analyses were performed using R version 4.3.1 (R Foundation for Statistical Computing) using libraries: tidyverse, ggplot2, gtsummary and summarytools [14].

### Ethical consideration

Prior to commencing this study, ethical approval was sought and obtained from the ethics committees of the University of Rwanda College of Medicine and Health Sciences and the University Teaching Hospital of Kigali (CMHS/IRB N°224/CMHS IRB/2021; CHUK/IRB Ref.: EC/CHUK/094/2021).

## Results

### Overview of the cohort

A total of 122 eligible patients were enrolled in the cohort. The median age was 55 years and 57% (n = 70) were female. The majority (93%, n = 114) had at least one comorbidity, with the most prevalent being diabetes (44%, n = 51), hypertension (34%, n = 42), chronic kidney disease (CKD stage 3 or above, 17%, n = 20) and HIV (17%, n = 19; Table 1).

**Table 1:** Overview of the cohort

Characteristic	N = 122^a^
Age (years)	55 (39, 67)
Gender	
Female	70 (57%)
Male	52 (43%)
At least 1 comorbidity	114 (93%)
*HIV status*	
Negative	96 (83%)
Positive	19 (17%)
Diabetes	51 (44%)
Hypertension	42 (34%)
Chronic kidney disease (CKD ≥ 3)	20 (17%)
Stroke	13 (11%)
Malignancy	13 (11%)
Malnutrition	8 (6.6%)
Cirrhosis	4 (3.5%)

^a^Median (IQR); n (%)

### Overview of infections:

The most common infection diagnoses were urinary tract infection (n = 36, 30%), pneumonia (n = 30, 25%) and bacteraemia (n = 21, 17%), of which 10 were catheter-related (8.2%; see Table 2). Approximately half the patients were enrolled due to fevers that began prior to or within 72 h of admission (community-onset; n = 64, 52%) with the remainder developing fevers more than 72 h into their admission (hospital-onset; n = 58, 48%).

**Table 2:** Overview of infection syndromes

Overview of infections	N = 122^a^
*Onset of fever*	
Prior to/on admission	64 (52%)
During hospitalization	58 (48%)
*Infection diagnosis*	
Urinary tract infection	36 (30%)
Pneumonia	30 (25%)
Primary bacteraemia	11 (9.0%)
Catheter-related bloodstream infection	10 (8.2%)
Diabetic foot infection	9 (7.4%)
Cellulitis	4 (3.3%)
Intra-abdominal abscess	4 (3.3%)
Spontaneous bacterial peritonitis	4 (3.3%)
Empyema thoracis	4 (3.3%)
Aspiration pneumonia	2 (1.6%)
Infected chronic ulcer	2 (1.6%)
Soft tissue abscess	2 (1.6%)
Bacterial meningitis	1 (0.8%)
Central line infection (without bacteraemia)	1 (0.8%)
Lung abscess	1 (0.8%)
Typhoid fever	1 (0.8%)

^a^n (%)

Gram-negative organisms were most common (82%, n = 100/122), with *E. coli* accounting for 33% (n = 40) of all infections, followed by *Klebsiella pneumoniae* (30%, n = 36). Table 3 outlines the most common pathogens according to the site of culture.

**Table 3:** List of pathogens according to site of culture. A list of pathogens according to final diagnosis can also be found in the supplementary data (Additional file 1)

Organism	Source of culture								
	Overall, N = 122^a^	Urine^a^	Blood^a^	Sputum^a^	Pus or wound swab^a^	Tracheal aspirate^a^	Pleural fluid^a^	Ascites^a^	CSF^a^
*E. coli*	40 (33%)	23 (64%)	5 (23%)	2 (9.1%)	8 (36%)	1 (9.1%)	0 (0%)	1 (25%)	0 (0%)
*Klebsiella pneumoniae*	36 (30%)	7 (19%)	6 (27%)	12 (55%)	8 (36%)	3 (27%)	0 (0%)	0 (0%)	0 (0%)
*Staphylococcus aureus*	14 (11%)	1 (2.8%)	9 (41%)	1 (4.5%)	3 (14%)	0 (0%)	0 (0%)	0 (0%)	0 (0%)
*Acinetobacter* spp.	7 (5.7%)	1 (2.8%)	0 (0%)	2 (9.1%)	0 (0%)	3 (27%)	0 (0%)	1 (25%)	0 (0%)
*Pseudomonas aeruginosa*	6 (4.9%)	0 (0%)	0 (0%)	2 (9.1%)	0 (0%)	3 (27%)	1 (25%)	0 (0%)	0 (0%)
*Enterobacter* spp.	4 (3.3%)	2 (5.6%)	0 (0%)	2 (9.1%)	0 (0%)	0 (0%)	0 (0%)	0 (0%)	0 (0%)
*Providencia* spp.	3 (2.5%)	1 (2.8%)	0 (0%)	0 (0%)	0 (0%)	1 (9.1%)	0 (0%)	1 (25%)	0 (0%)
*Streptococcus pneumoniae*	3 (2.5%)	0 (0%)	0 (0%)	0 (0%)	0 (0%)	0 (0%)	2 (50%)	0 (0%)	1 (100%)
*Enterococcus* spp.	2 (1.6%)	1 (2.8%)	0 (0%)	0 (0%)	0 (0%)	0 (0%)	0 (0%)	1 (25%)	0 (0%)
*Streptococcus* spp.	2 (1.6%)	0 (0%)	1 (4.5%)	1 (4.5%)	0 (0%)	0 (0%)	0 (0%)	0 (0%)	0 (0%)
*Proteus mirabilis*	1 (0.8%)	0 (0%)	0 (0%)	0 (0%)	1 (4.5%)	0 (0%)	0 (0%)	0 (0%)	0 (0%)
*Proteus vulgaris*	1 (0.8%)	0 (0%)	0 (0%)	0 (0%)	1 (4.5%)	0 (0%)	0 (0%)	0 (0%)	0 (0%)
*Salmonella typhi*	1 (0.8%)	0 (0%)	1 (4.5%)	0 (0%)	0 (0%)	0 (0%)	0 (0%)	0 (0%)	0 (0%)
*Serratia* spp.	1 (0.8%)	0 (0%)	0 (0%)	0 (0%)	1 (4.5%)	0 (0%)	0 (0%)	0 (0%)	0 (0%)
*Streptococcus pyogenes*	1 (0.8%)	0 (0%)	0 (0%)	0 (0%)	0 (0%)	0 (0%)	1 (25%)	0 (0%)	0 (0%)

^a^n (%)

### Antibiotic exposure

**Fig. 1:**
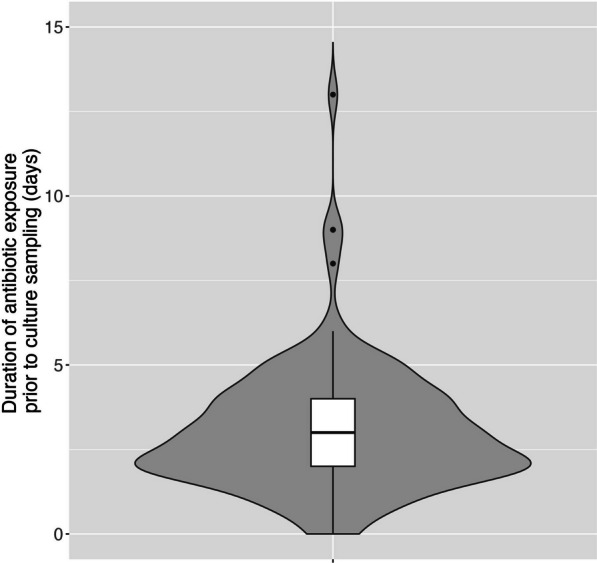
Composite violin and box plot showing the duration of exposure to antibiotics prior to culture sampling. This figure only includes prior inpatient exposure to the particular antibiotic being received at the time of the culture

The median duration of fever at the time of culture sampling was 7 days (IQR 5–10 days). By the time of culture sampling, 98% of patients (n = 119) were already receiving an antibiotic, with a median duration of exposure to that antibiotic of 3 days (IQR 2–4 days; Fig. 1). The most commonly prescribed antibiotic at the time of culture was ceftriaxone (66%, n = 80). Table 4 shows a detailed breakdown of antimicrobial exposure at the time of culturing.

**Table 4:** Proportion of patients receiving each antibiotic at the time of the culture being taken

Antibiotic exposure prior to culture	N = 122^a^
Ceftriaxone	80 (66%)
Metronidazole	33 (27%)
Meropenem	18 (15%)
Cefotaxime	14 (11%)
Cloxacillin	13 (11%)
Doxycycline	9 (7.4%)
Vancomycin	6 (4.9%)
Co-amoxiclav	5 (4.1%)
Ciprofloxacin	2 (1.6%)
Azithromycin	2 (1.6%)
Ampicillin	1 (0.8%)

^a^n (%)

### Antimicrobial susceptibility results

The rates of resistance amongst enteric Gram-negative pathogens of the *Enterobacterales* order (n = 87) were high, with only 28% of those tested (19/69) being susceptible to ceftriaxone, 45% (27/60) susceptible to gentamicin and 66% (40/61) susceptible to piperacillin-tazobactam. Reliable susceptibility was only seen to amikacin (92%, 68/74) and imipenem (90%, 64/71). Regarding possible oral antimicrobials, susceptibility of the *Enterobacterales* to ciprofloxacin was 44% (7/16), to co-amoxiclav was 2.5% (2/80) and to co-trimoxazole was 0% (0/8). Amongst the other Gram-negatives, high rates of carbapenem resistance were seen in *Acinetobacter* species, with only 20% (1/5) susceptible to imipenem. Table 5 provides a detailed breakdown of antimicrobial susceptibility according to organism.

**Table 5:** Proportion of organisms susceptible to each antimicrobial tested. Less prevalent organisms have been grouped by class and missing data are not presented here. Full organism-specific data, including missing values, are available in the supplementary data (Additional file 2)

Susceptibility	N	*E. coli*, N = 40^a^	*Klebsiella pneumoniae*, N = 36^a^	Other *Enterobacterales*, N = 11^a^	*Pseudomonas aeruginosa*, N = 6^a^	*Acinetobacter* spp., N = 7^a^	*Staphylococcus aureus*, N = 14^a^	*Streptococcus* spp., N = 6^a^	*Enterococcus* spp., N = 2^a^
Ceftriaxone	86	6 (19%)	9 (31%)	4 (50%)	1 (33%)	1 (20%)	0 (0%)	1 (25%)	0 (0%)
Amikacin	87	32 (91%)	25 (89%)	11 (100%)	6 (100%)	5 (71%)	0 (NA%)	0 (NA%)	0 (NA%)
Imipenem	83	31 (97%)	27 (84%)	6 (86%)	5 (83%)	1 (20%)	0 (NA%)	0 (0%)	0 (NA%)
Ceftazidime	37	2 (15%)	2 (15%)	1 (50%)	2 (40%)	0 (0%)	0 (NA%)	0 (NA%)	0 (NA%)
Polymyxin B	48	13 (81%)	12 (67%)	5 (71%)	3 (100%)	3 (75%)	0 (NA%)	0 (NA%)	0 (NA%)
Piperacillin-tazobactam	70	19 (68%)	17 (65%)	4 (57%)	4 (100%)	2 (40%)	0 (NA%)	0 (NA%)	0 (NA%)
Gentamicin	72	12 (46%)	11 (42%)	4 (50%)	2 (33%)	1 (25%)	2 (100%)	0 (NA%)	0 (NA%)
Ciprofloxacin	20	3 (30%)	4 (67%)	0 (NA%)	1 (100%)	0 (0%)	2 (100%)	0 (NA%)	0 (NA%)
Co-amoxiclav	84	1 (2.7%)	1 (3.1%)	0 (0%)	0 (0%)	0 (0%)	0 (NA%)	0 (0%)	1 (100%)
Cefotaxime	40	7 (44%)	3 (21%)	2 (40%)	0 (0%)	0 (0%)	0 (NA%)	2 (100%)	0 (NA%)
Cloxacillin	1	0 (NA%)	0 (NA%)	0 (NA%)	0 (NA%)	0 (NA%)	0 (NA%)	1 (100%)	0 (NA%)
Clindamycin	21	0 (NA%)	0 (NA%)	0 (NA%)	0 (NA%)	0 (NA%)	9 (69%)	5 (83%)	0 (0%)
Doxycycline	2	0 (NA%)	0 (NA%)	0 (NA%)	0 (NA%)	0 (NA%)	1 (100%)	1 (100%)	0 (NA%)
Vancomycin	20	0 (NA%)	0 (NA%)	0 (NA%)	0 (NA%)	0 (NA%)	13 (100%)	5 (100%)	2 (100%)
Penicillin	19	0 (NA%)	0 (NA%)	0 (NA%)	0 (NA%)	0 (NA%)	1 (7.1%)	2 (50%)	0 (0%)
Chloramphenicol	31	3 (75%)	9 (90%)	4 (67%)	1 (33%)	0 (0%)	4 (80%)	0 (NA%)	0 (NA%)
Amoxicillin	5	0 (NA%)	0 (NA%)	0 (NA%)	0 (NA%)	0 (NA%)	0 (0%)	1 (33%)	0 (NA%)
Oxacillin	14	0 (NA%)	0 (NA%)	0 (NA%)	0 (NA%)	0 (NA%)	12 (100%)	0 (0%)	0 (0%)
Erythromycin	13	0 (NA%)	0 (NA%)	0 (NA%)	0 (NA%)	0 (NA%)	6 (60%)	3 (100%)	0 (NA%)
Cefuroxime	3	0 (NA%)	0 (NA%)	0 (NA%)	0 (NA%)	0 (NA%)	2 (100%)	0 (NA%)	0 (0%)
Tetracycline	5	0 (NA%)	0 (NA%)	0 (NA%)	0 (NA%)	0 (NA%)	1 (33%)	1 (100%)	0 (0%)
Pefloxacin	11	2 (40%)	1 (50%)	1 (100%)	0 (NA%)	0 (0%)	0 (0%)	0 (NA%)	0 (NA%)
Nitrofurantoin	3	1 (33%)	0 (NA%)	0 (NA%)	0 (NA%)	0 (NA%)	0 (NA%)	0 (NA%)	0 (NA%)
Mecillinam	1	0 (0%)	0 (NA%)	0 (NA%)	0 (NA%)	0 (NA%)	0 (NA%)	0 (NA%)	0 (NA%)
Co-trimoxazole	8	0 (0%)	0 (0%)	0 (0%)	0 (NA%)	0 (NA%)	0 (NA%)	0 (NA%)	0 (NA%)

^a^n (% of isolates tested for susceptibility to that antimicrobial)

N = number of isolates tested for susceptibility to that antibiotic

Of the 14 *Staphylococcus aureus* isolates grown, none were identified as methicillin-resistant *Staphylococcus aureus* (MRSA; missing data for two isolates). All staphylococcal, streptococcal and enterococcal isolates had preserved susceptibility to vancomycin (100%, n = 20, missing data for two isolates).

Patients with a hospital-onset of fever were significantly more likely to have an isolate that was resistant to a 3rd-generation cephalosporin (OR 3.87, 95% CI 1.63–9.88, *p* = 0.003).

### Clinical outcomes and the association with resistance

Overall, 106 patients (87%) were discharged with resolution of fever; the remaining 16 patients (13%) died during their admission. The median length of hospital stay was 17 days (IQR 10–27 days).

Of the 119 patients who received antimicrobial therapy prior to culture sampling, sixty-six patients (55%) were initially prescribed an antimicrobial regime to which their subsequent culture isolate was resistant. Discordance between empirical antibiotic choice and subsequent culture result was strongly associated with in-hospital mortality (OR 6.87, 95% CI 1.80–45.1, *p* = 0.014).

## Discussion

This study, set in the adult medical wards and ICU of a tertiary referral hospital in Kigali, Rwanda, found high rates of antimicrobial resistance, particularly among Gram-negative organisms. Critically, however, 98% of the patients were already antibiotic-exposed at the time of culture sampling, introducing a potential selection bias towards isolation of resistant organisms and highlighting the limitations of routine laboratory data for antimicrobial resistance (AMR) surveillance in this setting. There were significant delays in culture taking, with a median duration of antibiotic exposure of 3 days prior to culture sampling. Unsurprisingly, discordant antibiotic therapy was significantly associated with in-hospital mortality.

The rates of resistance presented here are broadly consistent with those reported previously in Rwanda. A retrospective study from 2017 to 2018, which looked at 341 bacteraemias from the same referral hospital, found an identical rate of ceftriaxone resistance of 73% amongst the tested Gram-negative organisms (97 of 132 isolates) [6]. Worryingly, however, the rates of imipenem resistance were much higher in our cohort compared to this cohort from only 4 years earlier (15% vs. 1%). By contrast, MRSA rates were higher in the earlier cohort, with a prevalence of 33% (23 out of 69 isolates), and there were 3 cases of vancomycin-resistant *Staphylococcus aureus* (VRSA; 3.7%, 3 out of 78 tested isolates). This contrasts with no MRSA or VRSA seen in our study. Of note, however, the earlier cohort was hospital-wide, included only bloodstream infections and 25% of the cohort were patients under 14 years of age. Importantly, their data were only laboratory-based and so there was no corresponding clinical information about antibiotic exposure at the time of culture.

Another study from 2019 at the same institution enrolled 647 adult patients with suspected infection and financed microbiological cultures for them according to the presumed site of infection [7]. They identified 323 positive cultures, with a similar predominance of Gram-negative organisms (88% compared to 82% in our cohort). They also found a similar rate of ceftriaxone resistance amongst Gram-negatives (76% vs. 73% in our cohort), but lower rates of carbapenem resistance (4% compared to 15% in our cohort). They were able to perform limited testing for ESBL-production and found this to be the main mechanism of cephalosporin resistance (71% of the 92 tested Enterobacteriaceae). Similar to Habyarimana et al*.*, they found rates of MRSA of 32%. Sixty-five percent of their patients had been exposed to antibiotics in the 30 days prior to enrolment but they did not collect data on the nature, timing or duration of that exposure, nor on how many patients received antibiotics after enrolment but prior to culture.

A retrospective study from a different referral hospital in Kigali examined resistance patterns amongst 5296 laboratory isolates from 2009 to 2013 [9]. They found lower rates of resistance compared to our study: 64% of *E. coli* and 75% of *Klebsiella pneumoniae* isolates were resistant to co-amoxiclav (compared 97.3% and 96.9% respectively in our cohort), and only 25% of *E. coli* were resistant to cefuroxime (compared to 81% resistance to ceftriaxone in our cohort). Similar to our study, they found low rates of MRSA and VRE (only 2.2% and 0.6% respectively). Again, however, as a laboratory-based study, they did not report on antibiotic exposure of patients prior to these cultures being taken.

There appears to be a clear temporal trend towards worsening AMR amongst Gram-negative organisms isolated in referral hospitals in Rwanda, with a worrying increase in carbapenem resistance in our cohort. Indeed, an older study from 2009 found only 38% ceftriaxone resistance amongst inpatient *E. coli* isolates from urine (compared to 81% in our cohort) [15]. Although testing capacity for resistance mechanisms is limited, it is likely that extended-spectrum beta-lactamase (ESBL) production accounts for a significant proportion of this worsening resistance. A study looking at carriage of ESBL-producing Enterobacteriaceae amongst patients and caregivers in a referral hospital in southern Rwanda identified high rates of ESBL-carriage (50% of patients and 37% of caregivers at admission, increasing to 65% and 47% at discharge) [8].

Nonetheless, our study highlights the significant challenge of using routine laboratory samples to infer the broader burden of AMR in our setting. The majority of existing literature in Rwanda, and indeed many low-income countries, provides little or no data on the clinical context of the cultures being analysed. By linking clinical and laboratory data, we found that 98% of our patients had received antibiotic therapy prior to the culture being taken, with 66% specifically exposed to ceftriaxone and a median prior exposure of 3 days. In this context, it is not surprising to find a high level of ESBL-producing organisms amongst culture results. Patients with susceptible infections are either not being cultured (due to rapid clinical improvement with empiric therapy) or their cultures are more likely to be negative due to prior effective antibiotic exposure (with the exception perhaps of those with a deep-focus of infection and inadequate source control). This selection bias means that resistant infections may be over-represented among positive laboratory cultures, particularly compared to high-income settings where microbiological culture is more routinely performed prior to antibiotic administration and at lower-level health facilities. This phenomenon is increasingly recognised as a limitation of passive, laboratory-based AMR surveillance [2, 16, 17]. In our institution, availability of blood culture bottles is inconsistent and doctors have to check with the laboratory before requesting. This discourages routine requesting of blood cultures unless a patient is deteriorating on first-line therapy.

Evidence-based antimicrobial stewardship programmes and national antimicrobial guidelines rely entirely on the quality of data used to inform them. The existing data from Rwanda would support the need for a carbapenem and/or amikacin for all unwell patients admitted to teaching hospitals in Rwanda with a possible Gram-negative infection. However, our findings on the delay to culture and significant selection bias from prior antibiotic exposure should give policymakers pause for thought. The risk of over-estimating AMR is significant, particularly in the context of the already increasing prevalence of carbapenem resistance seen in our cohort. There is a need for enhanced AMR surveillance, culturing antibiotic-naïve patients at all levels of healthcare (community, primary and secondary, not just in tertiary referral hospitals). This would not only provide data for better-informed national policies, but also support clinicians in narrowing the spectrum of antibiotic therapy in those with susceptible infections that are not being isolated in current practice.

Finally, the reported prevalence of MRSA in Rwanda varies considerably, ranging from 0 to 2% in our study and Carroll et al*.*, to 32–33% in Habyarimana et al*.* and Sutherland et al., and even 82% in one study from 2013 [6, 7, 9, 10]. Whilst variation in patient cohort selection may account for some differences, it is possible that rates of MRSA may be miscalculated due to faulty laboratory materials or over-estimated where mixed staphylococcal cultures are not correctly identified (disc diffusion tests may be misread as resistant in mixed infections as coagulase-negative *Staphylococci* are usually methicillin-resistant). Further work is needed to delineate this issue, as most patients do not receive empiric MRSA coverage in Rwanda and the introduction of such practice would pose a significant risk in the absence of therapeutic drug monitoring for glycopeptides and could drive further resistance.

Our study has several limitations, most notably being single-centre, single-specialty and relying on a single non-automated laboratory for culture interpretation. Nonetheless, these real-world clinical culture results are all that are available to policymakers in Rwanda and the detailed clinical and prescribing data presented here highlight the challenges of relying on such data to infer the true prevalence of AMR.

## Conclusion

Overall, this study has identified an alarming rate of AMR in medical inpatients at a referral hospital in Rwanda. Resistance amongst Gram-negative organisms was of particular concern, with high rates of cephalosporin resistance and emerging carbapenem resistance. However, the cohort also had significant antibiotic exposure prior to culture sampling. This highlights the limitations of existing laboratory data in Rwanda, which are exclusively from patients in referral hospitals and may not be representative of the wider population. Future efforts should focus on understanding the prevalence of AMR in patients earlier in their pathway, starting with their first presentation to healthcare in community settings and prior to administration of antibiotics.

### Supplementary Information

**Additional file 1:** Organisms according to final diagnosis.

**Additional file 2:** Full susceptibility testing data by organism species, with missing values included.

## Data Availability

All aggregate data analysed during this study are included in this published article and its supplementary information files. Anonymised raw data may be available from the corresponding author upon reasonable request.
