# Supplementary material for: The prevalence and clinical context of antimicrobial resistance amongst medical inpatients at a referral hospital in Rwanda: a cohort study
Source: Antimicrob Resist Infect Control. 2024 Feb 22;13:22. doi: 10.1186/s13756-024-01384-7 (PMC10885367; doi:10.1186/s13756-024-01384-7)
Supplement: Supplementary file 1 — Additional file 1: Organisms according to final diagnosis. [file 13756_2024_1384_MOESM1_ESM.docx]

|  | **Final diagnosis** | | | | | | | | | | | | | | | | |
| --- | --- | --- | --- | --- | --- | --- | --- | --- | --- | --- | --- | --- | --- | --- | --- | --- | --- |
| **Organism** | **Overall**, N = 122*^1^* | **Urinary tract infection**, N = 36*^1^* | **Pneumonia**, N = 30*^1^* | **Primary bacteraemia**, N = 11*^1^* | **Catheter-related bloodstream infection**, N = 10*^1^* | **Diabetic foot infection**, N = 9*^1^* | **Cellulitis**, N = 4*^1^* | **Empyema thoracis**, N = 4*^1^* | **Intra-abdominal abscess**, N = 4*^1^* | **Spontaneous bacterial peritonitis**, N = 4*^1^* | **Aspiration pneumonia**, N = 2*^1^* | **Soft tissue abscess**, N = 2*^1^* | **Infected chronic ulcer**, N = 2*^1^* | **Bacterial meningitis**, N = 1*^1^* | **Central line infection (without bacteraemia)**, N = 1*^1^* | **Lung abscess**, N = 1*^1^* | **Typhoid fever**, N = 1*^1^* |
| *E. coli* | 40 (33%) | 23 (64%) | 3 (10%) | 3 (27%) | 2 (20%) | 3 (33%) | 3 (75%) | 0 (0%) | 1 (25%) | 1 (25%) | 0 (0%) | 0 (0%) | 1 (50%) | 0 (0%) | 0 (0%) | 0 (0%) | 0 (0%) |
| *Klebsiella pneumoniae* | 36 (30%) | 7 (19%) | 14 (47%) | 5 (45%) | 1 (10%) | 3 (33%) | 0 (0%) | 0 (0%) | 3 (75%) | 0 (0%) | 0 (0%) | 1 (50%) | 1 (50%) | 0 (0%) | 0 (0%) | 1 (100%) | 0 (0%) |
| *Staphylococcus aureus* | 14 (11%) | 1 (2.8%) | 0 (0%) | 2 (18%) | 7 (70%) | 0 (0%) | 1 (25%) | 0 (0%) | 0 (0%) | 0 (0%) | 1 (50%) | 1 (50%) | 0 (0%) | 0 (0%) | 1 (100%) | 0 (0%) | 0 (0%) |
| *Acinetobacter* spp*.* | 7 (5.7%) | 1 (2.8%) | 5 (17%) | 0 (0%) | 0 (0%) | 0 (0%) | 0 (0%) | 0 (0%) | 0 (0%) | 1 (25%) | 0 (0%) | 0 (0%) | 0 (0%) | 0 (0%) | 0 (0%) | 0 (0%) | 0 (0%) |
| *Pseudomonas aeruginosa* | 6 (4.9%) | 0 (0%) | 5 (17%) | 0 (0%) | 0 (0%) | 0 (0%) | 0 (0%) | 1 (25%) | 0 (0%) | 0 (0%) | 0 (0%) | 0 (0%) | 0 (0%) | 0 (0%) | 0 (0%) | 0 (0%) | 0 (0%) |
| *Enterobacter* spp*.* | 4 (3.3%) | 2 (5.6%) | 2 (6.7%) | 0 (0%) | 0 (0%) | 0 (0%) | 0 (0%) | 0 (0%) | 0 (0%) | 0 (0%) | 0 (0%) | 0 (0%) | 0 (0%) | 0 (0%) | 0 (0%) | 0 (0%) | 0 (0%) |
| *Providencia* spp*.* | 3 (2.5%) | 1 (2.8%) | 1 (3.3%) | 0 (0%) | 0 (0%) | 0 (0%) | 0 (0%) | 0 (0%) | 0 (0%) | 1 (25%) | 0 (0%) | 0 (0%) | 0 (0%) | 0 (0%) | 0 (0%) | 0 (0%) | 0 (0%) |
| *Streptococcus pneumoniae* | 3 (2.5%) | 0 (0%) | 0 (0%) | 0 (0%) | 0 (0%) | 0 (0%) | 0 (0%) | 2 (50%) | 0 (0%) | 0 (0%) | 0 (0%) | 0 (0%) | 0 (0%) | 1 (100%) | 0 (0%) | 0 (0%) | 0 (0%) |
| *Enterococcus* spp*.* | 2 (1.6%) | 1 (2.8%) | 0 (0%) | 0 (0%) | 0 (0%) | 0 (0%) | 0 (0%) | 0 (0%) | 0 (0%) | 1 (25%) | 0 (0%) | 0 (0%) | 0 (0%) | 0 (0%) | 0 (0%) | 0 (0%) | 0 (0%) |
| *Streptococcus* spp*.* | 2 (1.6%) | 0 (0%) | 0 (0%) | 1 (9.1%) | 0 (0%) | 0 (0%) | 0 (0%) | 0 (0%) | 0 (0%) | 0 (0%) | 1 (50%) | 0 (0%) | 0 (0%) | 0 (0%) | 0 (0%) | 0 (0%) | 0 (0%) |
| *Proteus mirabilis* | 1 (0.8%) | 0 (0%) | 0 (0%) | 0 (0%) | 0 (0%) | 1 (11%) | 0 (0%) | 0 (0%) | 0 (0%) | 0 (0%) | 0 (0%) | 0 (0%) | 0 (0%) | 0 (0%) | 0 (0%) | 0 (0%) | 0 (0%) |
| *Proteus vulgaris* | 1 (0.8%) | 0 (0%) | 0 (0%) | 0 (0%) | 0 (0%) | 1 (11%) | 0 (0%) | 0 (0%) | 0 (0%) | 0 (0%) | 0 (0%) | 0 (0%) | 0 (0%) | 0 (0%) | 0 (0%) | 0 (0%) | 0 (0%) |
| *Salmonella typhi* | 1 (0.8%) | 0 (0%) | 0 (0%) | 0 (0%) | 0 (0%) | 0 (0%) | 0 (0%) | 0 (0%) | 0 (0%) | 0 (0%) | 0 (0%) | 0 (0%) | 0 (0%) | 0 (0%) | 0 (0%) | 0 (0%) | 1 (100%) |
| *Serratia* spp*.* | 1 (0.8%) | 0 (0%) | 0 (0%) | 0 (0%) | 0 (0%) | 1 (11%) | 0 (0%) | 0 (0%) | 0 (0%) | 0 (0%) | 0 (0%) | 0 (0%) | 0 (0%) | 0 (0%) | 0 (0%) | 0 (0%) | 0 (0%) |
| *Streptococcus pyogenes* | 1 (0.8%) | 0 (0%) | 0 (0%) | 0 (0%) | 0 (0%) | 0 (0%) | 0 (0%) | 1 (25%) | 0 (0%) | 0 (0%) | 0 (0%) | 0 (0%) | 0 (0%) | 0 (0%) | 0 (0%) | 0 (0%) | 0 (0%) |
| *^1^*n (%) | | | | | | | | | | | | | | | | | |

**Supplementary Data 1:** Organisms according to final diagnosis.
