# Supplementary material for: The prevalence and clinical context of antimicrobial resistance amongst medical inpatients at a referral hospital in Rwanda: a cohort study
Source: Antimicrob Resist Infect Control. 2024 Feb 22;13:22. doi: 10.1186/s13756-024-01384-7 (PMC10885367; doi:10.1186/s13756-024-01384-7)
Supplement: Supplementary file 2 — Additional file 2: Full susceptibility testing data by organism species, with missing values included. [file 13756_2024_1384_MOESM2_ESM.docx]

| **Susceptibility** | **N** | | **Overall**,  N = 122*^1^* | | ***Acinetobacter* spp.**,  N = 7*^1^* | | ***E. coli***,  N = 40*^1^* | | ***Enterobacter* spp.**,  N = 4*^1^* | | ***Enterococcus* spp.**,  N = 2*^1^* | | ***Klebsiella pneumoniae***, N = 36*^1^* | | ***Proteus mirabilis***,  N = 1*^1^* | | ***Proteus vulgaris***,  N = 1*^1^* | | ***Providencia* spp.**,  N = 3*^1^* | | ***Pseudomonas aeruginosa***, N = 6*^1^* | | ***Salmonella typhi***,  N = 1*^1^* | | ***Serratia* spp.**,  N = 1*^1^* | | ***Staphylococcus aureus***,  N = 14*^1^* | | | ***Streptococcus* *pneumoniae***, N = 3*^1^* | | ***Streptococcus* *pyogenes***,  N = 1*^1^* | | ***Streptococcus* spp.**,  N = 2*^1^* | |  |
| --- | --- | --- | --- | --- | --- | --- | --- | --- | --- | --- | --- | --- | --- | --- | --- | --- | --- | --- | --- | --- | --- | --- | --- | --- | --- | --- | --- | --- | --- | --- | --- | --- | --- | --- | --- | --- |
| Ceftriaxone | 86 | | 22 (26%) | | 1 (20%) | | 6 (19%) | | 2 (67%) | | 0 (0%) | | 9 (31%) | | 0 (NA%) | | 0 (NA%) | | 0 (0%) | | 1 (33%) | | 1 (100%) | | 1 (100%) | | 0 (0%) | | | 0 (0%) | | 0 (0%) | | 1 (50%) | |  |
| Not tested |  | | 36 | | 2 | | 8 | | 1 | | 0 | | 7 | | 1 | | 1 | | 0 | | 3 | | 0 | | 0 | | 11 | | | 2 | | 0 | | 0 | |  |
| Amikacin | 87 | | 79 (91%) | | 5 (71%) | | 32 (91%) | | 4 (100%) | | 0 (NA%) | | 25 (89%) | | 1 (100%) | | 1 (100%) | | 3 (100%) | | 6 (100%) | | 1 (100%) | | 1 (100%) | | 0 (NA%) | | | 0 (NA%) | | 0 (NA%) | | 0 (NA%) | |  |
| Not tested |  | | 35 | | 0 | | 5 | | 0 | | 2 | | 8 | | 0 | | 0 | | 0 | | 0 | | 0 | | 0 | | 14 | | | 3 | | 1 | | 2 | |  |
| Imipenem | 83 | | 70 (84%) | | 1 (20%) | | 31 (97%) | | 1 (50%) | | 0 (NA%) | | 27 (84%) | | 1 (100%) | | 0 (NA%) | | 3 (100%) | | 5 (83%) | | 1 (100%) | | 0 (NA%) | | 0 (NA%) | | | 0 (0%) | | 0 (NA%) | | 0 (NA%) | |  |
| Not tested |  | | 39 | | 2 | | 8 | | 2 | | 2 | | 4 | | 0 | | 1 | | 0 | | 0 | | 0 | | 1 | | 14 | | | 2 | | 1 | | 2 | |  |
| Ceftazidime | 37 | | 7 (19%) | | 0 (0%) | | 2 (15%) | | 0 (NA%) | | 0 (NA%) | | 2 (15%) | | 0 (NA%) | | 1 (100%) | | 0 (0%) | | 2 (40%) | | 0 (NA%) | | 0 (NA%) | | 0 (NA%) | | | 0 (NA%) | | 0 (NA%) | | 0 (NA%) | |  |
| Not tested |  | | 85 | | 3 | | 27 | | 4 | | 2 | | 23 | | 1 | | 0 | | 2 | | 1 | | 1 | | 1 | | 14 | | | 3 | | 1 | | 2 | |  |
| Polymyxin B | 48 | | 36 (75%) | | 3 (75%) | | 13 (81%) | | 3 (100%) | | 0 (NA%) | | 12 (67%) | | 0 (0%) | | 0 (0%) | | 1 (100%) | | 3 (100%) | | 0 (NA%) | | 1 (100%) | | 0 (NA%) | | | 0 (NA%) | | 0 (NA%) | | 0 (NA%) | |  |
| Not tested |  | | 74 | | 3 | | 24 | | 1 | | 2 | | 18 | | 0 | | 0 | | 2 | | 3 | | 1 | | 0 | | 14 | | | 3 | | 1 | | 2 | |  |
| Piperacillin-tazobactam | 70 | | 46 (66%) | | 2 (40%) | | 19 (68%) | | 2 (67%) | | 0 (NA%) | | 17 (65%) | | 1 (100%) | | 1 (100%) | | 0 (0%) | | 4 (100%) | | 0 (NA%) | | 0 (NA%) | | 0 (NA%) | | | 0 (NA%) | | 0 (NA%) | | 0 (NA%) | |  |
| Not tested |  | | 52 | | 2 | | 12 | | 1 | | 2 | | 10 | | 0 | | 0 | | 1 | | 2 | | 1 | | 1 | | 14 | | | 3 | | 1 | | 2 | |  |
| Gentamicin | 72 | | 32 (44%) | | 1 (25%) | | 12 (46%) | | 1 (33%) | | 0 (NA%) | | 11 (42%) | | 0 (0%) | | 1 (100%) | | 1 (50%) | | 2 (33%) | | 0 (NA%) | | 1 (100%) | | 2 (100%) | | | 0 (NA%) | | 0 (NA%) | | 0 (NA%) | |  |
| Not tested |  | | 50 | | 3 | | 14 | | 1 | | 2 | | 10 | | 0 | | 0 | | 1 | | 0 | | 1 | | 0 | | 12 | | | 3 | | 1 | | 2 | |  |
| Ciprofloxacin | 20 | | 10 (50%) | | 0 (0%) | | 3 (30%) | | 0 (NA%) | | 0 (NA%) | | 4 (67%) | | 0 (NA%) | | 0 (NA%) | | 0 (NA%) | | 1 (100%) | | 0 (NA%) | | 0 (NA%) | | 2 (100%) | | | 0 (NA%) | | 0 (NA%) | | 0 (NA%) | |  |
| Not tested |  | | 102 | | 6 | | 30 | | 4 | | 2 | | 30 | | 1 | | 1 | | 3 | | 5 | | 1 | | 1 | | 12 | | | 3 | | 1 | | 2 | |  |
| Co-amoxiclav | 84 | | 3 (3.6%) | | 0 (0%) | | 1 (2.7%) | | 0 (0%) | | 1 (100%) | | 1 (3.1%) | | 0 (0%) | | 0 (0%) | | 0 (0%) | | 0 (0%) | | 0 (0%) | | 0 (0%) | | 0 (NA%) | | | 0 (NA%) | | 0 (0%) | | 0 (NA%) | |  |
| Not tested |  | | 38 | | 6 | | 3 | | 0 | | 1 | | 4 | | 0 | | 0 | | 0 | | 5 | | 0 | | 0 | | 14 | | | 3 | | 0 | | 2 | |  |
| Cefotaxime | 40 | | 14 (35%) | | 0 (0%) | | 7 (44%) | | 0 (0%) | | 0 (NA%) | | 3 (21%) | | 1 (100%) | | 1 (100%) | | 0 (0%) | | 0 (0%) | | 0 (NA%) | | 0 (NA%) | | 0 (NA%) | | | 2 (100%) | | 0 (NA%) | | 0 (NA%) | |  |
| Not tested |  | | 82 | | 6 | | 24 | | 2 | | 2 | | 22 | | 0 | | 0 | | 2 | | 4 | | 1 | | 1 | | 14 | | | 1 | | 1 | | 2 | |  |
| Cloxacillin | 1 | | 1 (100%) | | 0 (NA%) | | 0 (NA%) | | 0 (NA%) | | 0 (NA%) | | 0 (NA%) | | 0 (NA%) | | 0 (NA%) | | 0 (NA%) | | 0 (NA%) | | 0 (NA%) | | 0 (NA%) | | 0 (NA%) | | | 0 (NA%) | | 0 (NA%) | | 1 (100%) | |  |
| Not tested | |  | | 121 | | 7 | | 40 | | 4 | | 2 | | 36 | | 1 | | 1 | | 3 | | 6 | | 1 | | 1 | | | 14 | | 3 | | 1 | | 1 | |
| Clindamycin | | 21 | | 14 (67%) | | 0 (NA%) | | 0 (NA%) | | 0 (NA%) | | 0 (0%) | | 0 (NA%) | | 0 (NA%) | | 0 (NA%) | | 0 (NA%) | | 0 (NA%) | | 0 (NA%) | | 0 (NA%) | | | 9 (69%) | | 3 (100%) | | 1 (100%) | | 1 (50%) | |
| Not tested | |  | | 101 | | 7 | | 40 | | 4 | | 0 | | 36 | | 1 | | 1 | | 3 | | 6 | | 1 | | 1 | | | 1 | | 0 | | 0 | | 0 | |
| Doxycycline | | 2 | | 2 (100%) | | 0 (NA%) | | 0 (NA%) | | 0 (NA%) | | 0 (NA%) | | 0 (NA%) | | 0 (NA%) | | 0 (NA%) | | 0 (NA%) | | 0 (NA%) | | 0 (NA%) | | 0 (NA%) | | | 1 (100%) | | 0 (NA%) | | 0 (NA%) | | 1 (100%) | |
| Not tested | |  | | 120 | | 7 | | 40 | | 4 | | 2 | | 36 | | 1 | | 1 | | 3 | | 6 | | 1 | | 1 | | | 13 | | 3 | | 1 | | 1 | |
| Vancomycin | | 20 | | 20 (100%) | | 0 (NA%) | | 0 (NA%) | | 0 (NA%) | | 2 (100%) | | 0 (NA%) | | 0 (NA%) | | 0 (NA%) | | 0 (NA%) | | 0 (NA%) | | 0 (NA%) | | 0 (NA%) | | | 13 (100%) | | 2 (100%) | | 1 (100%) | | 2 (100%) | |
| Not tested | |  | | 102 | | 7 | | 40 | | 4 | | 0 | | 36 | | 1 | | 1 | | 3 | | 6 | | 1 | | 1 | | | 1 | | 1 | | 0 | | 0 | |
| Penicillin | | 19 | | 3 (16%) | | 0 (NA%) | | 0 (NA%) | | 0 (NA%) | | 0 (0%) | | 0 (NA%) | | 0 (NA%) | | 0 (NA%) | | 0 (NA%) | | 0 (NA%) | | 0 (NA%) | | 0 (NA%) | | | 1 (7.1%) | | 1 (100%) | | 1 (100%) | | 0 (0%) | |
| Not tested | |  | | 103 | | 7 | | 40 | | 4 | | 1 | | 36 | | 1 | | 1 | | 3 | | 6 | | 1 | | 1 | | | 0 | | 2 | | 0 | | 0 | |
| Chloramphenicol | | 31 | | 21 (68%) | | 0 (0%) | | 3 (75%) | | 2 (100%) | | 0 (NA%) | | 9 (90%) | | 1 (100%) | | 0 (0%) | | 1 (100%) | | 1 (33%) | | 0 (0%) | | 0 (NA%) | | | 4 (80%) | | 0 (NA%) | | 0 (NA%) | | 0 (NA%) | |
| Not tested | |  | | 91 | | 4 | | 36 | | 2 | | 2 | | 26 | | 0 | | 0 | | 2 | | 3 | | 0 | | 1 | | | 9 | | 3 | | 1 | | 2 | |
| Amoxicillin | | 5 | | 1 (20%) | | 0 (NA%) | | 0 (NA%) | | 0 (NA%) | | 0 (NA%) | | 0 (NA%) | | 0 (NA%) | | 0 (NA%) | | 0 (NA%) | | 0 (NA%) | | 0 (NA%) | | 0 (NA%) | | | 0 (0%) | | 1 (33%) | | 0 (NA%) | | 0 (NA%) | |
| Not tested | |  | | 117 | | 7 | | 40 | | 4 | | 2 | | 36 | | 1 | | 1 | | 3 | | 6 | | 1 | | 1 | | | 12 | | 0 | | 1 | | 2 | |
| Oxacillin | | 14 | | 12 (86%) | | 0 (NA%) | | 0 (NA%) | | 0 (NA%) | | 0 (0%) | | 0 (NA%) | | 0 (NA%) | | 0 (NA%) | | 0 (NA%) | | 0 (NA%) | | 0 (NA%) | | 0 (NA%) | | | 12 (100%) | | 0 (NA%) | | 0 (NA%) | | 0 (0%) | |
| Not tested | |  | | 108 | | 7 | | 40 | | 4 | | 1 | | 36 | | 1 | | 1 | | 3 | | 6 | | 1 | | 1 | | | 2 | | 3 | | 1 | | 1 | |
| Erythromycin | | 13 | | 9 (69%) | | 0 (NA%) | | 0 (NA%) | | 0 (NA%) | | 0 (NA%) | | 0 (NA%) | | 0 (NA%) | | 0 (NA%) | | 0 (NA%) | | 0 (NA%) | | 0 (NA%) | | 0 (NA%) | | | 6 (60%) | | 2 (100%) | | 1 (100%) | | 0 (NA%) | |
| Not tested | |  | | 109 | | 7 | | 40 | | 4 | | 2 | | 36 | | 1 | | 1 | | 3 | | 6 | | 1 | | 1 | | | 4 | | 1 | | 0 | | 2 | |
| Cefuroxime | | 3 | | 2 (67%) | | 0 (NA%) | | 0 (NA%) | | 0 (NA%) | | 0 (0%) | | 0 (NA%) | | 0 (NA%) | | 0 (NA%) | | 0 (NA%) | | 0 (NA%) | | 0 (NA%) | | 0 (NA%) | | | 2 (100%) | | 0 (NA%) | | 0 (NA%) | | 0 (NA%) | |
| Not tested | |  | | 119 | | 7 | | 40 | | 4 | | 1 | | 36 | | 1 | | 1 | | 3 | | 6 | | 1 | | 1 | | | 12 | | 3 | | 1 | | 2 | |
| Tetracycline | | 5 | | 2 (40%) | | 0 (NA%) | | 0 (NA%) | | 0 (NA%) | | 0 (0%) | | 0 (NA%) | | 0 (NA%) | | 0 (NA%) | | 0 (NA%) | | 0 (NA%) | | 0 (NA%) | | 0 (NA%) | | | 1 (33%) | | 1 (100%) | | 0 (NA%) | | 0 (NA%) | |
| Not tested | |  | | 117 | | 7 | | 40 | | 4 | | 1 | | 36 | | 1 | | 1 | | 3 | | 6 | | 1 | | 1 | | | 11 | | 2 | | 1 | | 2 | |
| Pefloxacin | | 11 | | 4 (36%) | | 0 (0%) | | 2 (40%) | | 0 (NA%) | | 0 (NA%) | | 1 (50%) | | 0 (NA%) | | 0 (NA%) | | 1 (100%) | | 0 (NA%) | | 0 (NA%) | | 0 (NA%) | | | 0 (0%) | | 0 (NA%) | | 0 (NA%) | | 0 (NA%) | |
| Not tested | |  | | 111 | | 5 | | 35 | | 4 | | 2 | | 34 | | 1 | | 1 | | 2 | | 6 | | 1 | | 1 | | | 13 | | 3 | | 1 | | 2 | |
| Nitrofurantoin | | 3 | | 1 (33%) | | 0 (NA%) | | 1 (33%) | | 0 (NA%) | | 0 (NA%) | | 0 (NA%) | | 0 (NA%) | | 0 (NA%) | | 0 (NA%) | | 0 (NA%) | | 0 (NA%) | | 0 (NA%) | | | 0 (NA%) | | 0 (NA%) | | 0 (NA%) | | 0 (NA%) | |
| Not tested | |  | | 119 | | 7 | | 37 | | 4 | | 2 | | 36 | | 1 | | 1 | | 3 | | 6 | | 1 | | 1 | | | 14 | | 3 | | 1 | | 2 | |
| Mecillinam | | 1 | | 0 (0%) | | 0 (NA%) | | 0 (0%) | | 0 (NA%) | | 0 (NA%) | | 0 (NA%) | | 0 (NA%) | | 0 (NA%) | | 0 (NA%) | | 0 (NA%) | | 0 (NA%) | | 0 (NA%) | | | 0 (NA%) | | 0 (NA%) | | 0 (NA%) | | 0 (NA%) | |
| Not tested | |  | | 121 | | 7 | | 39 | | 4 | | 2 | | 36 | | 1 | | 1 | | 3 | | 6 | | 1 | | 1 | | | 14 | | 3 | | 1 | | 2 | |
| Co-trimoxazole | | 8 | | 0 (0%) | | 0 (NA%) | | 0 (0%) | | 0 (0%) | | 0 (NA%) | | 0 (0%) | | 0 (NA%) | | 0 (NA%) | | 0 (NA%) | | 0 (NA%) | | 0 (NA%) | | 0 (NA%) | | | 0 (NA%) | | 0 (NA%) | | 0 (NA%) | | 0 (NA%) | |
| Not tested | |  | | 114 | | 7 | | 36 | | 3 | | 2 | | 33 | | 1 | | 1 | | 3 | | 6 | | 1 | | 1 | | | 14 | | 3 | | 1 | | 2 | |
| *^1^*n (%) | | | | | | | | | | | | | | | | | | | | | | | | | | | |  |  |  |  |  |  |  |  |  |
| *N = number of isolates tested for susceptibility* | | | | | | | | | | | | | | | | | | | | | | | | | | | |  |  |  |  |  |  |  |  |  |

**Supplementary Data 2:** Full susceptibility testing data by organism species, with missing values included.
